# Distribution of Suicides in India: A Decadal Data Analysis (2011-2020): Areiba Arif

**DOI:** 10.1093/eurpub/ckac131.502

**Published:** 2022-10-25

**Authors:** A Arif

**Affiliations:** Public Policy, IIM Ahmedabad, Ahmedabad, India

## Abstract

**Background:**

India accounts for a significant proportion of suicide deaths globally. As per the Global Burden of Disease Study, India’s share of global suicides from 1990 to 2016 increased from 25·3% to 36·6% among women and 18·7% to 24·3% among men. However, medical factors are not the sole contributors to the suicide burden.

**Methods:**

I analyze decadal data (2011-2020) on suicides in India provided by National Crime Records Bureau. This data contains the distribution of suicidal deaths based on age groups, causes, professions, gender, and residence zones.

**Results:**

The analysis suggests that individuals aged ‘15-29 years’ were most affected (35.05%) by suicides, followed by those aged ‘30-44 years’ (32.61%). Family problems contribute to the highest burden of suicides among both these age groups. Such deaths can be attributed to personal reasons. Suicides due to dowry disputes are exclusive to women. Also, women report the highest instances of suicides due to marriage-related issues. While suicides due to drug abuse, bankruptcy, unemployment, poverty, and property disputes were mainly reported among males. In 2020, the rise in annual suicide rate was found to be highest among business persons (29.43%), especially tradesmen (49.9%) and vendors (26.11%) against other businesses (12.13%). Other professions which witnessed a steep rise in annual suicide rates in 2020 were agricultural laborers (17.90%) and daily wage earners (15.76%). This increase in suicide rates may be linked to economic reasons post lockdown imposition during the Covid-19 outbreak in 2020.

**Conclusions:**

Women commit suicide mainly due to marriage-related causes, while men are more vulnerable to suicide due to economic factors. Not all suicides can be traced back to diagnosed mental illnesses. A significant proportion of suicides are attributed to personal, economic, and social problems. The insights generated from this analysis can help identify the vulnerable groups and target the much-needed interventions.

**Key messages:**

• Suicide is a multifaceted problem involving various personal, economic, cultural, and social factors, besides medical reasons.

• Multidimensional strategies targeted at vulnerable groups could be potentially effective in curbing suicide rate.

